# Perceived barriers and facilitators to exercise in kidney transplant recipients: A qualitative study

**DOI:** 10.1111/hex.13423

**Published:** 2022-01-10

**Authors:** Roseanne E. Billany, Alice C. Smith, Clare Stevinson, Amy L. Clarke, Matthew P. M. Graham‐Brown, Nicolette C. Bishop

**Affiliations:** ^1^ Department of Cardiovascular Sciences University of Leicester Leicester UK; ^2^ John Walls Renal Unit University Hospitals of Leicester NHS Trust Leicester UK; ^3^ Department of Health Sciences University of Leicester Leicester UK; ^4^ School of Sport, Exercise and Health Sciences Loughborough University Loughborough UK; ^5^ Centre for Behavioural Medicine, UCL School of Pharmacy University College London London UK

**Keywords:** chronic kidney disease, exercise, kidney transplantation, qualitative research, semistructured interview

## Abstract

**Background:**

Exercise has the potential to attenuate the high levels of cardiovascular morbidity and mortality present in kidney transplant recipients (KTRs). Despite this, activity levels in KTRs remain low. The aim of this qualitative study was to explore the barriers and facilitators of exercise in KTRs.

**Methods:**

Thirteen KTRs (eight males; mean ± SD; age 53 ± 13 years; estimated glomerular filtration rate 53 ± 21 ml/min/1.73 m^2^) were recruited and completed semistructured one‐to‐one interviews at University Hospitals of Leicester NHS Trust. All KTRs were eligible if their kidney transplant was completed >12 weeks before interview and their consultant considered them to have no major contraindications to exercise. All interviews were audio recorded, transcribed verbatim and subject to framework analysis to identify and report themes.

**Results:**

Themes were organized into personal, behavioural and environmental factors based on social cognitive theory. Facilitators of exercise were largely internal: enjoyment, exercise for general health and health of the transplanted kidney and desire to maintain normality. Social interaction, support and guidance of healthcare professionals and goal setting were perceived as motivational. Harming the kidney, a lack of guidance, self‐motivation and accessibility were barriers to exercise.

**Conclusion:**

These results provide detailed insight into the development of interventions designed to increase physical activity in KTRs. They provide strong evidence that specific exercise guidelines are required for this population and that the healthcare system could have a key role in supporting KTRs to become more physically active. Interventions need to be multifaceted to appeal to the differing levels of support desired by KTRs.

**Patient or Public Contribution:**

KTRs were involved in the development of the interview topic guide to ensure all relevant topics were explored.

## 1 INTRODUCTION

Adopting a healthy lifestyle with exercise is recommended by the clinical practice guideline for the care of kidney transplant recipients (KTRs).^1^ It is estimated that less than one in three KTRs reach the minimum level of 150 min of moderate‐intensity physical activity per week as recommended by the World Health Organization.^2^ Cardiovascular disease (CVD) remains a leading cause of morbidity and mortality in KTRs, which has been associated with the elevated traditional and nontraditional risk factors present in this population.^3^ Exercise in the general population associates with a less deleterious CVD risk‐factor profile, less cardiovascular morbidity and mortality^4^ and a better quality of life.^5^ Although abundant empirical evidence is lacking for these associations in KTRs, positive effects of exercise have been reported,^6^ and it remains an important therapeutic choice in posttransplantation management.

Sánchez et al.^7^ surveyed common barriers to physical activity in KTRs, which included: lack of motivation (62%), preferring other things (47%), bad weather (47%), fatigue (46%) and health conditions (43%). Common facilitators were: feeling healthy (96%), wanting to feel better (93%), wanting to improve health (93%), wanting to enhance physical mobility (90%) and wanting increased strength (89%). Encouragement from healthcare providers (78%) was also a frequently endorsed facilitator. The majority of studies outlining the barriers and facilitators to exercise in KTRs are survey‐based. Studies that widen the focus to explore why and how these factors influence physical activity behaviour beyond a fixed response would provide valuable evidence to inform future practice. For example, what type of encouragement from healthcare providers serves as a facilitator to physical activity. This would allow identification of specific factors that could be drawn upon to design appropriately tailored guidelines and to develop and implement behaviour change programmes designed to promote increased activity in this unique population. The need for this detailed information has been highlighted and the importance of developing evidence‐based exercise guidelines and individually tailored exercise regimens based on the needs and resources of the individuals has been stressed.^8^

The aim of this study was to identify and explore the perceived barriers and facilitators to exercise in greater depth than the current literature provides. These results can be appropriately utilized in the development of future interventions addressing the low levels of physical activity in KTRs.

## 2 MATERIALS AND METHODS

### 2.1 Study design

This study was conducted under the constructivist paradigm allowing the formation of assumptions and construction of meaning to be drawn from the data.^9^ Social cognitive theory (SCT) was used as a conceptual framework to organize the data and to portray how the findings could be utilized in future intervention development. Data were collected using one‐to‐one semistructured interviews to explore perceptions and experiences of exercise. The wider study was approved by the NRES Committee East Midlands—Northampton (13/06/2012; UK NIHR Clinical Research Network Portfolio Study number: 12716 ‘QCKD’). The target minimum sample size was 12 with a maximum sample size of 15. This was with consideration to time restraints and resources with respect to gathering and analysing the data. This was in line with recommendations and evaluation of prior research by Francis et al.^10^ It is also in line with ‘typical’ sample sizes for phenomenological studies,^11^ which suggest that a sample size of 12 is likely to lead to saturation of the data.

### 2.2 Recruitment

Participants were recruited using a convenience sampling method from University Hospitals of Leicester NHS Trust (UHL) kidney outpatient clinics. Consultants screened clinic lists based on study inclusion/exclusion criteria. Eligible patients were approached after their clinical consultation and were given a patient information sheet. All patients who had received their kidney transplant >12 weeks were eligible unless their clinician deemed them to have a major contraindication to exercise. Although no physical exercise was required for this study, it was considered unsuitable to ask these participants exercise‐related questions. Exclusion criteria were: unstable angina or myocardial infarction during the previous 6 weeks, severe heart failure, severe chronic obstructive pulmonary disease, severe lower limb orthopaedic problems or severe lower limb neuromuscular disease.

### 2.3 Data collection

All one‐to‐one semistructured interviews were conducted by R. E. B. in a private room at UHL following written informed consent. Participants had no pre‐existing relationship with the researcher. An interview topic guide was developed and piloted with two KTRs. Adjustments were made in accordance with feedback before proceeding (see Supporting Information A). Interviews had two parts: (1) general exercise, and (2) high‐intensity interval training. This report focuses on (1) general exercise due to a large volume of data produced. Part 1 covered the following topics: general exercise attitudes; current exercise; benefits of exercise; negative elements of exercise; barriers to exercise; reasons for exercising; exercise benefits and drawbacks with a specific focus on being a KTR. Interviews lasted between 20 and 60 min. Probes were used to ensure rich detailed data. All interviews were recorded digitally, anonymized and professionally transcribed verbatim. All transcripts were read whilst simultaneously listening to audio files to ensure accuracy. All audio files and transcripts were imported into NVivo 11 (QSR International NVivo 11 Pro). Demographic and clinical data were extracted from medical records. Current weekly physical activity was descriptively obtained from interview data.

### 2.4 Data analysis

Data were analysed using framework analysis, providing a flexible pragmatic approach to explore a broad area of research without being bound to a particular epistemological position.^12^ The flexibility of framework analysis allowed for a complementary inductive and deductive approach to analysis. The inductive approach allowed us first to gain insights into the key barriers and drivers of exercise, which subsequently allowed us to identify a suitable theory, which could be used to apply a deductive approach to our analysis.^13^ Gale et al.^13^ have broken down the original five‐phase analysis^14^ into seven phases in the context of multidisciplinary health research, which were followed for the present analysis: (1) transcription, (2) familiarization with the interview, (3) coding, (4) developing a working analytical framework, (5) applying the analytical framework, (6) charting the data into the framework matrix and (7) interpreting the data. Initial coding of two transcripts was completed by two researchers (R. E. B. and C. S.) to ensure consistency of interpretation. A working analytical framework was agreed upon and applied to the remaining transcripts by R. E. B. Newly identified codes were added throughout the process and the framework was not finalized until the final transcript. Some of the themes presented can be identified as both barriers and facilitators to exercise. Patterns based on age, gender and exercise frequency were searched for in the data. No patterns were identified based on age or gender. Patterns based on exercise frequency are reported within the results.

The themes identified were compatible with SCT that provides a framework to explain how the interaction between personal, behavioural and environmental factors influence behaviour.^15^ SCT was utilized to inform the analysis and themes were categorized according to the three factors.

## 3 RESULTS

Thirty‐two KTR were invited to take part in the study. Thirteen were recruited and completed a one‐to‐one semistructured interview (participant characteristics are outlined in Table 1). Interviews ranged in duration between 19 and 59 min, with a mean duration of 34 min. Nineteen patients declined to participate or did not meet the inclusion/exclusion criteria. Patients were not required to give reasons for declining participation but, for those who did, the main reasons for decline (and for exclusion) were: lack of time, distance to travel, language barrier, burden of comorbidities and low transplant function. These results describe the perceived barriers and motivators of exercise in KTR in the context of SCT.

**Table 1:** Participant characteristics

Code (QCKDTx)	Gender	Age (years)	Ethnicity	eGFR (ml/min/1.73 m^2^)	Transplant time (years. months)	Type of transplant		Current weekly physical activity^a^
HI01	Male	36	White British	90	2.10	DBD		2 × weights sessions, 2 × running sessions, tai chi daily
HI02	Female	57	White British	90	36.1	DBD		Occasional walks (not always weekly)
HI03	Male	65	White British	48	0.7	DCD		Walking most days (normal pace)
HI04	Male	59	White British	30	24.4	DCD		3 × gym sessions, walking
HI05	Female	44	White British	60	15.9^b^	DBD		1 × weights class, 1 × interval/swimming session, 1 × 1 h run
HI06	Female	68	White British	47	3.4	Living non‐R		1 × gym session, regular walking (normal pace)
HI07	Male	42	White British	57	0.3	Living non‐R		~4 bike rides, walking
HI08	Male	73	Asian	41	8.9	DBD		Daily stretching and breathing exercises, daily 30 min walk (normal pace)
HI09	Female	48	White British	62	0.8^b^	DBD		2 × Body Combat class, occasional bike ride
HI10	Male	47	White British	26	7.4	Living R		Occasional walks (not always weekly)
HI11	Male	49	White British	52	2.8	Living R		Gym/swim at least 4 days
HI12	Female	32	White British	60	5.2	Living R		Occasional walks (not always weekly)
HI13	Male	63	White British	24	0.4	DCD		2–3 × gym sessions (mainly 2 currently)

Abbreviations: DBD, donation after brain stem death; DCD, donation after circulatory death; eGFR, estimated glomerular filtration rate; R, related.

^a^ Descriptive evaluation taken from interview transcripts.

^b^ Second transplant.

### 3.1 Personal factors

Four themes were identified relating to personal factors, each with several subthemes. Table 2 presents example quotations for each theme.

**Table 2:** Personal factors example quotations

Theme	Subtheme(s)	Example quotations
Physical and mental benefits	General well‐being	‘I think it's maintaining a healthy, maintaining like a healthy life, healthy body, keeps your heart strong, lungs and just general health really’ (Male, 47)
	Improvements in specific health outcomes	‘And that's why it is very important to me because regular exercise as you know keeps the body in a good state so you don't, it combats getting over weight, keeps your cardiovascular system in good condition, it helps keep blood pressure down’ (Male, 36)
	Mental well‐being	‘And I think without that I think you'd just sit down and worry about the transplant, I think you'd just be sat there thinking about it all the time. It takes your mind off of that’. (Male, 63)
	Longevity of the new kidney	‘If exercise helps the kidney function and what have you with my body, then I'll do it’. (Male, 47)
	Stress relief	‘And especially if I get stressed because sometimes my job can be quite stressful and I just like going for a run because it kind of clears everything out and you are just focusing on your breathing and you are just hitting the road and its what's for me a great stress reliever’. (Male, 36)
Anxiety and self‐confidence	Harming the transplant	‘You have got this thing inside you and you are thinking if I do too much is it going to go wrong, is there going to be a problem with the graft. Especially when you have, as you know exercise is important for everyone, but when you have a transplant and you are so worried about oh I don't want to damage it, you can miss out on all the benefits of exercise if you are not exercising because you are worried you are going to damage yourself’. (Male, 36)
	Confidence in ability	‘Yeah, because especially now I get more like anxious about doing too much’. (Female, 32)
	Perception of age	‘And also last month I was 63, so I definitely wouldn't want to put my body through a punishing exercise regime every…’ (Male, 63)
	Restrictions	‘So I had to stop doing that and having been active I suppose that helped when I had the transplant I was physically quite fit really, but very quickly deteriorated as in I started putting on a little bit of weight. I really didn't start doing exercise until probably late 30s, early 40s’. (Male, 59)
	Heightened self‐awareness	‘It's the awareness of actually are you aching because it's not because of your transplant and I think participants can get particularly lazy because there's an excuse not to do something’. (Female, 44)
Self‐incentives	Normality	‘I've had a transplant to be normal like you lot so I can do it’. (HI09)
	Internal drive	‘I do get a bit down if I can't exercise, I get frustrated if I can't exercise. So I think there is a whole host of benefits to exercise and its very important to me’. (Male, 36)
	Curiosity	‘And after having the surgery I wanted to see what I could do, I wanted to see how far I could develop myself and push myself and that was a big part of it as well’. (Male, 36)
Self‐motivation	Lack of motivation	‘Yeah, it's just having that motivation’ (HI12)
	Desire to be motivated	‘I wish I had more motivation to do it, I tend to fits and starts when I do exercise’. (Female, 57)

#### 3.1.1 Physical and mental benefits (subthemes: General well‐being, improvements in specific health factors, musculoskeletal, mental well‐being, longevity of the new kidney and stress relief)

The majority of participants described the physical benefits of exercise to their general well‐being as their motivation. Those who did not exercise acknowledged the health benefits and expressed that they ‘should be doing it’. Preserving the longevity of the transplanted kidney was defined as highly important and exercise was suggested as a strong contributor to achieving this: ‘Not only because obviously it lowers levels but [I] also know damn well what effect it has on the kidney and ultimately I want it to last as long as possible…’ (Male, Age 59). Participants reported the role of exercise in reducing specific health risks, including elevated weight, high cholesterol and hypertension, with the desire to manage these being a significant incentive to becoming or staying active. Participants discussed the musculoskeletal benefits of exercise, including preserved mobility and increased muscular strength, and the importance of this to their kidney condition. Exercise was perceived to impact positively on mental well‐being by making participants ‘feel better’ and giving them mental ‘clarity’. Some participants defined exercise as a ‘stress‐relief’ and others as a way to ‘take their mind off’ their transplant and related worries.

#### 3.1.2 Anxiety and self‐confidence (subthemes: Harming the transplant, confidence in ability, perception of age, restrictions and heightened self‐awareness)

Participants held concerns about exercise harming their transplant, and felt they lacked knowledge of appropriate exercise and how hard they should be ‘pushing’ themselves. Concerns were felt to be greatest during the early stages of transplant, as participants ‘got used to it’ and as they were still healing. Some of the participants' feeling of anxiety stemmed from a ‘lack of self‐confidence’ in their appearance and ability to exercise: ‘I think it's just having that confidence to do things because I think going to the gym, unless I did it with someone else, I'd feel really anxious about going and just really like self‐conscious…’ (Female, Age 32). However, only two participants reported that their concerns prevented them from exercising. Martial arts and contact sports were frequently mentioned; some participants expressed disappointment that they could not participate in these activities posttransplant. One participant expressed that initial restrictions on lifting (e.g., heavy items, weight lifting) posttransplant elicited a natural tendency to restrict such activities for longer periods of time: ‘Some of it is perceived I think as well because obviously you get told you can't lift but that doesn't mean to say you can't lift light weight I'm sure. But, straight away you then start putting more restrictions on yourself and then obviously it just compounds’ (Male, Age 59). Participants reported a heightened sense of self‐awareness during exercise in terms of ‘listening to their bodies’. They reported being much more aware of feeling unwell and often felt they had an ‘internal dilemma’, debating if it was a normal illness, tiredness, or if it was related to the kidney. This heightened awareness led participants to be cautious about ‘doing too much’ and ‘feeling tired’ and was considered to impact on exercise behaviour as some would forgo exercise in favour of ‘resting their bodies’. Some participants stated that they had a much greater awareness of ‘normal’ exercise effects, such as increased blood pressure, heart rate and dehydration that caused them to ‘hold back’.

Contrary to these concerns, some participants believed they had confidence around exercising and they did not have any worries about their transplant: ‘No I always try and go as hard as I possibly can…’ (Male, Age 42).

Participants frequently discussed their age with respect to exercise. Age was sometimes perceived as a barrier to exercise, especially higher intensity. However, some participants believed that getting older was a motivation to be active.

#### 3.1.3 Self‐incentives (subthemes: Normality, internal drive, curiosity)

Participants who regularly exercised described an ‘internal need’ to do it. Some of this ‘drive’ to exercise came from knowledge of the benefits and a desire to maintain their health. Some reported adverse effects of not doing exercise; mainly ‘frustration’, ‘stress’, and ‘guilt’ for missing sessions: ‘And now exercise is an important part of my life. I feel if I miss a day I have to do it because I have to make up for it’. (Male, Age 49).

Having a ‘sense of normality’ was considered important to participants posttransplant: ‘You live your life as you pretty much would normally [with] common sense…’ (Male, Age 59). They described how they did not want to be regarded as a ‘patient’. Several participants believed that exercise was part of ‘normality’; however, for some participants ‘normality’ was being able to complete daily tasks without symptoms or assistance. Some participants believed the latter could be aided by exercise giving a perception of independence. More active participants reported finding it difficult to understand people, particularly other KTRs, who do not exercise: ‘I get annoyed with people who say [I] can't do it because I've got a bit of arthritis. I've had two new hips you can do it…you can walk, you can exercise’ (Female, Age 48). On the other hand, one participant did not describe themselves as ‘normal’ and referred to people without chronic kidney disease (CKD) as ‘normal’. This participant expressed a ‘lack of confidence’ in their knowledge of exercise and ability to exercise.

Participants described a sense of gratitude for receiving a kidney donation and a desire to explore their new capabilities: ‘It sounds cheesy, but I suppose I was just so grateful to be given this new lease of life, it's like a second chance almost and I felt well I want to make the very most of it. It's given me this opportunity to really make the most of my life and do all the things I had wanted to do and part of that is exercise as well’ (Male, Age 36). However, one participant described the battle to get back into exercise after transplant: ‘I mean I'm not enjoying it much at all at the moment because I'm trying to get back into condition and it's killing me’ (Male, Age 63).

#### 3.1.4 Self‐motivation (subthemes: Lack of motivation and desire to be motivated)

The least active participants described a lack of motivation towards exercise: ‘I don't know, I mean I just need to be more self‐motivated that's all, I think that's my biggest issue. If I had the motivation I could probably find the time, it's just I need some sort of a… sometimes need a push’ (Male, Age 47). Although these participants reported a lack of motivation towards exercise, they all described an awareness that they either ‘needed’ to do it, or they had a ‘desire’ to do it: ‘I mean I know it's good for you in lots of ways, and especially with some of the tablets I am on I need to exercise’ (Female, Age 57).

### 3.2 Environmental factors

Four themes related to environmental factors with various subthemes identified within each major theme. Table 3 presents example quotations for each theme.

**Table 3:** Environmental factors example quotations

Theme	Subtheme(s)	Example quotations
Social interaction	Social support	‘Saying that I do think it's nice if somebody goes with you, I have gone to places on my own because I have had to but I do feel if you can take a friend that's two of you getting the benefit and you are not alone’. (Female, 57)
	Family support	‘I'm so fortunate my whole family love exercise and obviously it shows now ones at a fitness and dance uni and the others doing a dance job’. (Female, 48)
	Accountability	‘…they'll, you know, persuade you to go and give you that motivation, which is always good because then when you do get there and you do do it, it does make you feel better’. (Female, 57)
	Expectations of other people	‘People were surprised, I mean the neighbours and that were coming up and saying oh Christ what's he doing he's had a transplant’. (Male, 65)
Physical environment	Weather	‘No. There are things about the weather that sometimes put me off exercising. In the middle of winter I don't like getting into a cold pool. So there's sort of temperature things I don't like’. (Male, 63)
	Accessibility	‘And its free, a big thing, because that's the other thing sometimes sports, gyms are so expensive aren't they’. (Female, 57)
Guidance and support	Organizational priorities	‘I've been coming here since 2004 and I've not got one complaint ever. But I think there should be another area then afterwards [exercise] where some people might not want to have that but at least you've got the option there’. (Male, 65)
	Lack of guidance	‘I don't really feel anything is given specific to transplant participants’. (Female, 44)
	Desire for guidance and informational needs	‘I would like having more guidance around…how much I should push myself if I want to’. (Male, 36)
Healthcare professionals influence	Importance of key healthcare providers	‘So I think what we need is if you say to the doctor well I have got this issue then somebody can tailor it to help, half the time you are just sent away and this is the exercise you have got to do. Some of us need a kick up the backside we need that encouragement to motivate us and know that within three months we are going to see some benefits’. (Female, 57)
	Professional support	‘Maybe they need someone specific that works with the dietician possibly and the doctors, so they are the recreational advisors’. (Female, 57)

#### 3.2.1 Social interaction (subthemes: Social support, family support, accountability and expectations of other people)

Whether or not participants considered exercise enjoyable, they regularly linked it with human interaction. Exercise was perceived by participants as a method of spending time with family and friends and making new friends. Having someone else to exercise with was considered motivating and encouraging: ‘So when you've got somebody then you've got companionship, you can do things between you rather than just going out and having to go to other classes’ (Female, Age 68). Participants also expressed not wanting to ‘let people down’ if they had planned to exercise together. One participant highlighted that not knowing any other transplant recipients who exercise was discouraging: ‘Well not for me, the problem is I don't know anybody else. So the lady I met the other day, the other one that introduced me, she's the first one really that I've met who actually talked to, and even then we didn't get to know one another very well. So whether anybody else is doing it, I have no idea’ (Female, Age 68).

Participants believed that other people's expectations (mainly strangers) were often different from their own, which were to regain ‘normality’ posttransplant. This portrayed a sense of a ‘lack of understanding’ about their situation: ‘…I had a kidney transplant. “Oh what and you're exercising?” That's their first words, that's their thoughts’ (Female, Age 48). However, these expectations that KTRs should not exercise were also expressed by other transplant recipients who were perhaps less confident in their abilities to exercise.

#### 3.2.2 Physical environment (subthemes: Weather and accessibility)

The physical environment was believed by participants to be important in determining exercise enjoyment. ‘Bad weather’ was often perceived as a barrier to being more active: ‘Not really, I know there is room for improvement, but summer will be coming…Sometimes the weather, you know if it's not very good outside you are not feeling motivated to go out and do anything’ (Female, Age 57). Accessibility was highlighted as a strong influencer of exercise behaviour. Participants felt that local facilities were off‐putting due to the high cost: ‘Also it's expensive to go and then you've got to join and then you've got to then go because if you don't go 3 times a week you're not getting your benefit out of paying for that. So then it becomes a chore because it's a got to go and then it's not enjoyable’ (Female, Age 68).

#### 3.2.3 Guidance and support (subthemes: Organizational priorities, lack of guidance, desire for guidance and informational needs)

Participants highly regarded the care they received throughout the whole transplant process. However, exercise advice and guidance was not viewed as a priority of the NHS by participants and some thought that it should be included in posttransplant care: ‘It's like you get so much about all your medication, all of the physical things in terms of, you know, like taking medication and just things like that, but in terms of your physical health I don't think you get…’ (Female, Age 32). Participants viewed doctors as the person they saw just for the ‘medical side of things’, mainly medication and symptoms.

More than half of the participants stated that they did not receive any specific advice or guidance around exercise. Those who did receive guidance reported receiving only ‘general advice’: ‘No‐one has…he just said to me you need to do more exercise’ (Male, Age 65). Two participants felt that they were supported and were provided with some specific advice. Although one explained how they had many more questions surrounding exercise limitations. Participants expressed a real desire for exercise guidance. This included advice on the benefits of exercise and different levels and types of support: ‘I would like having more guidance around…how much I should push myself if I want to’ (Male, Age 36). Participants expressed the desire for three different types of guidance. Firstly, standardized: ‘I think if it could be written at different levels for people who are at different capacities’ (Male, Age 63). Some participants who already exercised before transplant felt that this would be enough to support them back into exercise and help them know that they were doing the right things. Secondly, prescriptive (individualized): ‘…prescriptive exercise after transplant. I think that would be beneficial’ (Female, Age 44). Some participants preferred individually prescribed exercise that was personalized to them that they could work on between hospital appointments. Thirdly, supervised: ‘…just to get you into it and just feel supported with it’ (Female, Age 32). Several participants expressed a desire for supervised sessions, both individual and group. They felt that these would be beneficial to help them gain confidence and reassurance that what they are doing is not going to harm the kidney in any way. The least active participants discussed this type of guidance more. It was not expected that this supervision would be long‐term.

#### 3.2.4 Healthcare professionals influence (subthemes: Importance of key healthcare providers and professional support)

Overall participants expressed the importance of their healthcare provider as a factor in supporting and facilitating exercise behaviours. They perceived needing support and encouragement to start or return to exercise. Those with less exercise experience appeared to require greater support. Those who did exercise before transplant placed more importance on the healthcare provider providing accurate information on what they were able to do. In one case, lack of guidance directly resulted in limited exercise participation: ‘So I think it would be a good thing definitely to have, yeah, some sort of support afterwards with getting into exercise or getting back into exercise. And I think that's probably why I've never done it, because I've never really had that’ (Female, Age 32). Participants expressed some frustration about the advice and guidance given around exercise and creatinine levels. They described receiving different advice from different doctors that ultimately left them feeling ‘confused’: ‘You get different opinions from different doctors you really do’ (Female, Age 48). Some participants explained how the lack of detailed guidance has left them feeling confused and anxious about ‘knowing their exercise limits’.

### 3.3 Behavioural factors

Two themes were identified as behavioural factors with underlying subthemes. Table 4 presents example quotations for each theme.

**Table 4:** Behavioural factors example quotations

Theme	Subtheme(s)	Example quotations
Goal setting	Setting goals	‘I was doing three all the time before my transplant and now I'm getting back into the swing of it I'm usually only doing two. But my aim is to do three’. (Male, 65)
	Self‐management	
		‘I know I'm on a downwards trajectory now, I can sort of manage myself, aiming towards a goal of being fit rather than aiming to be fit for the transplant which is what I was doing before’. (Male, 42)
	Tracking improvements	‘So within 6 weeks of me going thinking I need to improve my fitness, my body toned up, I lost weight and obviously my fitness went [up]’. (Female, 48)
	Achievement	‘But I think once you have done it you feel the benefits after however long then you are probably more likely to either continue with something like that or take a step further and just go running or cycling or whatever’. (Female, 57)
Exercise preference	Activities	‘I think that sometimes if you think of exercise as in the gym or classes it can become tedious and you don't want to do it…I'd sooner do the walking and getting out and about and gardening’. (Female, 68)

#### 3.3.1 Goal setting (subthemes: Setting goals, self‐management, tracking improvements and achievement)

Setting goals was perceived by participants to be a motivator for continuing exercise. A structured approach was defined as a key ‘aid’ to exercise achievement: ‘I suppose just having structure, that's the biggest aid I have. And again going back to how much exercise has taught me about setting goals and achieving them and setting stress targets but also setting, doing it with baby steps’ (Male, Age 36). Those who exercised explained how they had specific aims in mind and strived to complete them and that being able to self‐manage was a contributor to increased confidence. Goals were largely autonomous, but one participant reported a preference for a professional to set goals: ‘…you maybe not be doing the right form of exercise for what you want or might be doing the right exercise but the wrong way’ (Male, Age 47). Observing improvements to functional and clinical outcomes after fulfilling aims was considered a motivating factor for participants to engage in sustained exercise.

#### 3.3.2 Exercise preference (subthemes: Activities)

Participants highlighted that their individual exercise preferences had a big impact on enjoyment which in turn seemed to influence continued exercise behaviours. A big factor in this was the location with many participants favouring exercise that was outdoors with ‘views’ and ‘fresh air’. Participants perceived this as a more ‘pleasurable’ experience: ‘Yeah it's much more pleasurable being in the countryside than exercising at home, cycling's just strolling through nice places for me really’ (Male, Age 42). Many participants reported walking as their preferred activity as it could be easily fitted into daily life. Some participants described how they preferred exercise classes as these were more structured and motivational. Several participants described their ‘housework’ and general chores as their way of being active. The gym was not perceived by many participants as a favourable exercise environment; often it was described as ‘boring’.

## 4 DISCUSSION

This qualitative research identifies perceived barriers and facilitators of exercise in KTRs. A recent study identified that approximately only 27% of KTRs are sufficiently active for health.^2^ Results of this study provide a more detailed understanding of the challenges experienced by KTRs in the context of increasing physical activity levels and provide some factors that may be utilized to promote an active lifestyle.

Fear of injuring the transplanted graft, insecurity with the body, and body signals have been previously postulated as a reason for lower levels of reported physical activity in KTRs.^8^, ^16^ Whilst this was present in the current study, participants expressed that a lack of guidance and a fear of the unknown were contributing factors. In solid organ transplant recipients (SOTR), lack of expertise of healthcare professionals was reported as a barrier to physical activity and support from professionals was identified as a strong facilitator.^16^ This is not isolated to transplant recipients; the positive impact that healthcare professionals can have on patient behaviours including physical activity is widely known.^17^ Improved education of lifestyle self‐management in CKD was identified as a need in the ‘Kidney Health: Delivering Excellence’ report.^18^ However, participants discussed feeling as though exercise was not a priority of the healthcare service, which is akin to previous findings in a CKD population.^19^ The latest ‘Management of Kidney Transplant Recipients by General Nephrologists’ curriculum states, in a section about weight control and exercise, that KTRs should be routinely and frequently counselled on the benefits of exercise.^20^ However, physicians are not routinely trained in exercise and physical activity prescription and formal referral pathways for rehabilitation do not exist for KTRs. Physicians who exercise regularly are more likely to counsel their patients to exercise and inadequate knowledge and experience is a barrier to counselling.^21^ A lack of evidence‐based exercise guidelines for KTRs is likely a factor in why there is suboptimal patient counselling from healthcare professionals, as well as a lack of time whilst providing essential care. Out of 34 Canadian physicians, only 18% were confident in performing physical activity counselling to their SOTR; lack of exercise guidelines was cited as one of the main barriers (53%).^22^ Specific guideline development has been identified as a future research priority^23^ and, healthcare professionals should have a key role in the development and implementation of these guidelines.^24^

All participants expressed a desire for more exercise guidance and advice, even those who had received some guidance appeared to have unanswered questions. Participants discussed three types of guidance: standardized, prescriptive and supervised. Standardized written guidance was favoured by those who already exercise and was perceived as sufficient information to aid them in feeling confident about appropriate exercise. Other participants desired more prescriptive exercise, which has been previously reported.^25^ Participants did not wish to have generic guidelines but instead preferred individualized regimens personalized by ability (not necessarily supervised). This was expressed by participants who were keen to set goals. Finally, supervised sessions were discussed mainly by the least active participants who felt that these sessions would give them confidence in their ability and reassure them that nothing they were doing would negatively impact the new kidney. Well‐established supervised exercise programmes have been identified as one of the most likely reasons why heart and lung transplant recipients exhibit greater physical activity levels than KTRs.^24^

A key influencer of exercise behaviours was social interaction. The relationship between social support and exercise has been linked to many theoretical perspectives: SCT,^15^ Theory of Planned Behaviour^26^ and Self‐Determination Theory.^27^ Social support (instrumental, emotional or informational) is thought to increase self‐efficacy and induce perceived behavioural control, which facilitates physical activity adherence and maintenance, especially if positive intentions are formed.^28^ Family support and inclusion was highly valued by participants as was exercise with friends. Exercising with someone or receiving encouragement to exercise was defined previously as an exercise strategy by KTRs.^25^ One participant expressed that not knowing any other KTRs who performed exercise resulted in feelings of isolation. This is supported by the findings of Clarke et al.,^19^ who found that participants expressed a desire to attend CKD‐specific exercise sessions, which would provide a safe environment. Sharing experiences and the support derived from fellow patients was beneficial during supervised rehabilitation in other chronic disease/pain management groups.^29^, ^30^

Self‐efficacy (confidence in one's ability to complete a particular behaviour) and self‐regulation (the control of one's behaviour through planning, setting goals and self‐monitoring) are thought to be fundamental cognitive factors influencing behaviour change within SCT. The least active participants described a lack of self‐confidence in their ability to exercise. Previous research has shown that fear of movement posttransplantation is related to low levels of physical activity and is strongly mediated by low self‐efficacy.^31^ Interventions should focus on improving self‐efficacy to foster positive and sustained exercise behaviours. Setting goals and monitoring progress was perceived as motivational by participants in this study and in others.^16^, ^19^ Self‐monitoring has been shown to be effective in improving physical activity in patients with CVD.^32^

Although this study has provided new insights into a wide range of exercise determinants in KTRs, some limitations are acknowledged. It is possible that this was a ‘self‐selecting’ group of KTRs who had an interest in exercise. Participant characteristics show that several participants were engaging in regular physical activity and therefore responses may not have captured the barriers to exercise experienced by those who are largely inactive. Nineteen participants declined to participate in the study. Interestingly, reasons for declining study participation,^33^ as mentioned above, are factors that also influence physical activity levels, which might explain why those volunteering were quite physically active overall. The results therefore may not capture all of the potential barriers to exercise. Future similar studies would benefit from capturing higher numbers of inactive KTRs.

The sample was not as diverse as intended; the participants of this study were almost all of a White British ethnicity. UHL cares for an ethnically diverse population of kidney patients and therefore some bias may have occurred during recruitment, potentially due to a language barrier with the researcher. Given that perceptions and barriers to exercise can bear cultural differences,^33^ future research should strive to include participants from a range of cultural backgrounds.

Some participants were less than 1‐year posttransplant and described themselves as ‘getting back into exercise’. Generally, during the year after transplant physical activity levels initially drop but then gradually increase above pretransplant levels.^34^ As participants gained more confidence in their ability to exercise, they may have changed their perceptions of exercise and their exercise behaviours. Inactive participants' views and perceptions may also change with increasing time posttransplant as they become accustomed to living with a transplant and resolve any medical issues that commonly arise during the initial months. Conversely, there were participants who had been transplanted for far longer (up to 24 years). There may have been differences in the type of posttransplant care and advice given recently compared with over a decade ago. Lifestyle has become a bigger focus in medicine over the past decade and continues to be a topic of debate amongst healthcare professionals and policy makers.^35^

In summary, KTRs reported some exercise barriers comparable to the general population and some specifically related to transplantation. In general, there was a positive attitude towards exercise and a desire to exercise, suggesting a potential positive uptake of specific evidence‐based exercise guidelines and research‐informed behaviour change interventions. There is a need for healthcare services to incorporate exercise into routine care addressing the complex and multiple needs of KTRs and a need to address the limiting factors of lack of time and knowledge for physical activity counselling amongst healthcare professionals. Further research will be fundamental in strengthening the body of evidence reporting the efficacy and effectiveness of exercise to support healthcare professionals in exercise counselling, the development of specific exercise guidelines and behaviour change interventions.

## CONFLICT OF INTERESTS

The authors declare that there are no conflict of interests.

## AUTHOR CONTRIBUTIONS

R. E. B. drafted the manuscript. A. C. S. is the grant holder and was responsible for the research idea, study design and management. N. C. B. and C. S. were involved in supervision. Each author contributed important intellectual content during manuscript revision and accepts responsibility for the overall work.

## Supporting information

Supporting information.

## Data Availability

Derived data supporting the findings of this study are available from the corresponding author N. C. B. on request.
