# Supplementary material for: Perceived barriers and facilitators to exercise in kidney transplant recipients: A qualitative study
Source: Health Expect. 2022 Jan 10;25(2):764–74. doi: 10.1111/hex.13423 (PMC8957725; doi:10.1111/hex.13423)
Supplement: Supplementary file 1 — Supporting information. [file HEX-25--s001.docx]

Appendix A: Interview Topic Guide

Version 1

**Introduction**

“Firstly welcome to the interview and thank you very much for agreeing to speak with us. The reason for this interview and the reason why we have asked you to take part for us is that we wish to understand more about exercise from the perspective of a kidney transplant recipient and also your thoughts and opinions about a specific type of exercise a little bit later on. It is important because there is limited information directly from the thoughts, views and opinions of patients.

I would like to remind you that anything you say here is anonymous. Your name or any personal details will not be mentioned in any reports produced following this interview. We will be using a digital recorder to record the conversation because it is difficult for us to write down everything you say accurately whilst also giving you full attention and listening to what you have to say.”

Do you have any questions before we start?

**Interview**

1. What are your thoughts about exercise?
   *What is it about exercise that you enjoy/dislike? Tell me about a time that you enjoyed exercise? In what ways does it help you as a kidney transplant patient? Tell me about a time, if any, that you felt exercise had a negative consequence?*
2. What do you think are the benefits of exercise?

*Do you think there are any benefits of exercise specific to people with a kidney transplant?*

1. What do you think are the negatives about exercise?

*Do you think there are any negatives of exercise specific to people with a kidney transplant?*

1. What does regular exercise mean to you?

*For example, if you exercised three times a week, would you say that is regular exercise for you?*

1. What exercise do you do or take part in? (This might be whilst commuting, specific activities, sports, walking etc.)

*How does exercise make you feel? Can you explain your reasons for exercising? Has having a kidney transplant influenced or changed the way that you exercise?
Can you explain the reasons for not exercising/not exercising more often? Are there reasons relating to your kidney transplant? Is there anything that might encourage you to exercise/exercise more often? Do you think you would exercise if you could attend supervised sessions? Are there any experiences in your past that have influenced this?*

1. Are you happy with your current level of exercise?

*If yes: What makes you happy about the amount of exercise that you take part in?*

*If no: Can you explain why you are not happy? Is there anything that might help you address this? Do you think that your kidney transplant prevents you from addressing this?*

1. What do you understand by the term “high intensity interval training”?

*You may have heard it referred to as “HIIT” training.*

*If no: move to definition*

*If yes: Where have you heard about it? What are your thoughts about it?*

***Definition***

***High intensity interval training is a form of interval training where there are short periods of maximal or near maximal effort followed by periods of less intense effort (also known as recovery periods). It is a form of cardiovascular exercise. Usually high intensity interval training refers to exercise performed on a bike or exercise bike and the term ‘sprint interval training’ refers to running whereby the periods of maximal or near maximal effort are sprinting and the recovery periods of less intense effort are jogging. High intensity interval training usually involves a warm up, several repetitions of maximal or near maximal effort followed by recovery and then finally a cool down. Examples of this might be:***

1. ***A 5 minute warm up followed by 6-10 repetitions of 30 second maximal or near maximal effort followed by 30 second recoveries. Ending with a 5 minute cool down.***
2. ***A 5 minute warm up followed by 4 repetitions of 4 minute maximal or near maximal effort followed by 3 minute recoveries. Ending with a 5 minute cool down.***

*Further questions after definition,*

- *Who do you think high intensity interval training is for?*
- *Who do you think high intensity interval training is not for?*

*Do you think clinical patients can take part in high intensity interval training?*

- *What do you think the negatives or problems surrounding high intensity interval training are or might be? Are there any specific ones relating to kidney transplant recipients?*
- *What do you think the positives or advantages surrounding high intensity interval training are? Are there any specific ones relating to kidney transplant recipients?*

1. Have you ever done any high intensity interval training?
   *If yes: What did you do? What encouraged you to do this? What did you think of it? How did it make you feel? How did it compare to other exercise that you might have done before? Was it supervised or unsupervised?*

*If no: Can you explain the reasons for not taking part in any high intensity interval training?*

1. How would you feel about being asked to do high intensity interval training?

*Is there anything about high intensity interval training that appeals to you? Is there anything about high intensity interval training that puts you off? What might encourage you to take part? Would you prefer it to be supervised or non-supervised (in your own time)? Are there any general concerns that you might have about it? Are there any concerns specific to your kidney transplant that you might have?*

1. What are your thoughts about high intensity interval training in comparison to ‘traditional’ aerobic exercise? (For example, 45 minutes continuous cycling at about 60% of your maximal effort)

*Do you think there are disadvantages over traditional aerobic exercise? Do you think there are advantages over traditional aerobic exercise?*

*Would your opinion differ if you were told that research has suggested that high intensity interval training gives better benefits in terms of improvements in fitness and factors related to diabetes and heart disease risk?*

*Would this make you more or less likely to take part in high intensity interval training than traditional aerobic exercise? What are your reasons for this?*

1. Is there anything that you would like to add or anything that you think we have missed during the interview?

Interview Topic Guide Version 2

**Introduction**

“Firstly welcome to the interview and thank you very much for agreeing to speak with us. The reason for this interview and the reason why we have asked you to take part for us is that we wish to understand more about exercise from the perspective of a kidney transplant recipient and also your thoughts and opinions about a specific type of exercise a little bit later on. It is important because there is limited information directly from the thoughts, views and opinions of patients.

I would like to remind you that anything you say here is anonymous. Your name or any personal details will not be mentioned in any reports produced following this interview. We will be using a digital recorder to record the conversation because it is difficult for us to write down everything you say accurately whilst also giving you full attention and listening to what you have to say.”

Do you have any questions before we start?

**Interview**

1. What are your thoughts about exercise?
   *What is it about exercise that you enjoy/dislike? Tell me about a time that you enjoyed exercise? In what ways does it help you as a kidney transplant patient? Tell me about a time, if any, that you felt exercise had a negative consequence?*
2. What do you think are the benefits of exercise?

*Do you think there are any benefits of exercise specific to people with a kidney transplant?*

1. What do you think are the negatives about exercise?

*Do you think there are any negatives of exercise specific to people with a kidney transplant?*

1. What does regular exercise mean to you?

*For example, if you exercised three times a week, would you say that is regular exercise for you?*

1. What exercise do you do or take part in? (This might be whilst commuting, specific activities, sports, walking etc.)

*How does exercise make you feel? Can you explain your reasons for exercising? Has having a kidney transplant influenced or changed the way that you exercise?
Can you explain the reasons for not exercising/not exercising more often? Are there reasons relating to your kidney transplant? Is there anything that might encourage you to exercise/exercise more often? Do you think you would exercise if you could attend supervised sessions? Are there any experiences in your past that have influenced this?*

1. What advice were you given about exercise after having your transplant?

*Did you feel like this was enough? Did this influence your exercise?*

*If no advice, what advice would you like to see?*

1. Are you happy with your current level of exercise?

*If yes: What makes you happy about the amount of exercise that you take part in?*

*If no: Can you explain why you are not happy? Is there anything that might help you address this? Do you think that your kidney transplant prevents you from addressing this?*

1. What do you understand by the term “high intensity interval training”?

*You may have heard it referred to as “HIIT” training.*

*If no: move to definition*

*If yes: Where have you heard about it? What are your thoughts about it?*

***Definition***

***High intensity interval training is a form of interval training where there are short periods of maximal or near maximal effort followed by periods of less intense effort (also known as recovery periods). It is a form of cardiovascular exercise. Usually high intensity interval training refers to exercise performed on a bike or exercise bike and the term ‘sprint interval training’ refers to running whereby the periods of maximal or near maximal effort are sprinting and the recovery periods of less intense effort are jogging. High intensity interval training usually involves a warm up, several repetitions of maximal or near maximal effort followed by recovery and then finally a cool down. Examples of this might be:***

1. ***A 5 minute warm up followed by 6-10 repetitions of 30 second maximal or near maximal effort followed by 30 second recoveries. Ending with a 5 minute cool down.***
2. ***A 5 minute warm up followed by 4 repetitions of 4 minute maximal or near maximal effort followed by 3 minute recoveries. Ending with a 5 minute cool down.***

*Further questions after definition,*

- *Who do you think high intensity interval training is for?*
- *Who do you think high intensity interval training is not for?*

*Do you think clinical patients can take part in high intensity interval training?*

- *What do you think the negatives or problems surrounding high intensity interval training are or might be? Are there any specific ones relating to kidney transplant recipients?*
- *What do you think the positives or advantages surrounding high intensity interval training are? Are there any specific ones relating to kidney transplant recipients?*

1. Have you ever done any high intensity interval training?
   *If yes: What did you do? What encouraged you to do this? What did you think of it? How did it make you feel? How did it compare to other exercise that you might have done before? Was it supervised or unsupervised?*

*If no: Can you explain the reasons for not taking part in any high intensity interval training?*

1. How would you feel about being asked to do high intensity interval training?

*Is there anything about high intensity interval training that appeals to you? Is there anything about high intensity interval training that puts you off? What might encourage you to take part? Would you prefer it to be supervised or non-supervised (in your own time)? Are there any general concerns that you might have about it? Are there any concerns specific to your kidney transplant that you might have?*

1. What are your thoughts about high intensity interval training in comparison to ‘traditional’ aerobic exercise? (For example, 45 minutes continuous cycling at about 60% of your maximal effort)

*Do you think there are disadvantages over traditional aerobic exercise? Do you think there are advantages over traditional aerobic exercise?*

*Would your opinion differ if you were told that research has suggested that high intensity interval training gives better benefits in terms of improvements in fitness and factors related to diabetes and heart disease risk?*

*Would this make you more or less likely to take part in high intensity interval training than traditional aerobic exercise? What are your reasons for this?*

1. Is there anything that you would like to add or anything that you think we have missed during the interview?
